# The Need to Distinguish between “Lapse” and “Relapse”

**DOI:** 10.1007/s40614-025-00436-6

**Published:** 2025-02-14

**Authors:** Katherine R. Brown

**Affiliations:** Sorenson Center for Clinical Excellence, 6405 Old Main Hill, Logan, UT 84321 USA

**Keywords:** Reemergence of problem behavior, Lapse, Relapse, Treatment

## Abstract

Over the last decade, behavior analysts have become increasingly interested in relapse and have used the term to describe the reemergence of problem behavior following successful reductions achieved during clinical intervention. Nonetheless, some elements of the current terminology may interfere with behavior analysts’ efforts to examine, predict, and mitigate the conditions that lead to the reemergence of severe problem behavior in clinical settings. The current article (1) describes how the term “relapse” has been used in the field of behavior analysis and fails to capture more nuanced effects observed in the reemergence of problem behavior; (2) suggests use of the terms “lapse” and “relapse” to capture these distinct effects; and (3) outlines the positive impact enhanced technological distinction between the concept of “lapse” and “relapse” could have on clinical research and practice.

The assessment and treatment of severe problem behavior, which includes functional analysis to identify variables maintaining problem behavior (Iwata et al., 1994) and function-based interventions, is often highly efficacious. Several large-*n* studies have shown function-based interventions often reduce problem behavior by 90% or greater (e.g., Greer et al., 2016; Phillips et al., 2017; Rooker et al., 2013). Despite the high efficacy of behavioral interventions, problem behavior frequently reemerges following successful intervention (e.g., Briggs et al., 2018; Mitteer et al., 2022; Muething et al., 2021). The reemergence of problem behavior threatens the long-term maintenance of treatment effects for problem behavior and can have several deleterious effects, including injury to the client or others and readmission to specialized behavioral health settings. Given the serious and often detrimental effects, there has been an increased focus on understanding conditions that lead to the reemergence of problem behavior.

The reemergence of problem behavior following successful intervention has commonly been referred to as relapse in applied behavior analytic literature (e.g., Lieving et al., 2004; Kimball et al., 2023; Neely et al., 2024; Pritchard et al., 2014; Wathen & Podlesnik, 2018). The concept of relapse has provided behavior analysts with a useful way to describe and examine the variables that influence the reemergence of problem behavior. Nevertheless, some elements of the current terminology may interfere with behavior analysts’ efforts to examine, predict, and mitigate the reemergence of problem behavior. One purpose of the current article is to describe how the concept of relapse has been used in the field of applied behavior analysis and how it fails to capture more nuanced effects of problem behavior re-emerging following intervention. A second purpose of this article is to suggest the use of alternative terms, lapse and relapse, to capture these nuanced differences and describe how the concepts of “lapse” and “relapse” are often related, yet distinct. A final purpose is to outline the positive impact that greater distinction between lapse and relapse phenomena could have on research and clinical practice for severe problem behavior.

## Making the Case There are Different Forms of “Relapse”

Researchers often use the term “relapse” to refer to the *temporary* reemergence of problem behavior under direct observation in tightly controlled conditions (cf. Kimball et al., 2023; Neely et al., 2024). This increased research focus on “relapse” has led to the identification and defining features of several conditions under which problem behavior may temporarily reemergence and threaten the long-term maintenance of severe behavior treatment effects, such as resurgence, renewal, reinstatement, and spontaneous recovery (cf. Kimball et al., 2023; Neely et al., 2024; Wathen & Podlesnik, 2018). Although each of these forms are distinct from one another, commonalities include that each involves a *transient* increase in problem behavior that can be remedied by continued implementation of an efficacious treatment. For example, resurgence is a temporary increase in problem behavior due to a worsening of reinforcement conditions (Lattal et al., 2017) and commonly occurs during reinforcement schedule thinning (Briggs et al., 2018; Falligant et al., 2022). Likewise, renewal is a temporary increase in problem behavior due to a change in stimulus context (Muething et al., 2020) and commonly occurs when treatment is implemented in a novel setting or with a novel implementer (Mitteer et al., 2022; Muething et al., 2020). Although the conditions that lead to the reemergence of problem behavior across these two examples are different (worsening of reinforcement conditions versus stimulus change), commonalities are the *transient or temporary* reemergence of problem behavior that can be remedied with continued implementation of the efficacious treatment. For example, Briggs et al. conducted a retrospective consecutive case series (CCCS) of 25 applications of reinforcement schedule thinning and observed the reemergence of problem behavior (i.e., resurgence) in 76% of applications (19 of 25). However, in all cases, problem behavior reemerged for a short period of time and then decreased following continued implementation of the treatment leading the researchers to conclude this was a transient phenomenon. As another example, Muething et al. (2020) conducted a retrospective CCCS of 67 cases in which context changes occurred (e.g., change in implementer, setting) and observed the reemergence of problem behavior (i.e., renewal) in 67.2% of cases (45 of 67). Following the context change, there was an initial increase in problem behavior that decreased across subsequent treatment sessions, once again highlighting the transient nature of this form of relapse given continued implementation of the efficacious treatment. Other known forms of relapse, such as spontaneous recovery and reinstatement, are also transient phenomena that decrease with continued implementation of the behavior intervention (Wathen & Podlesnik, 2018).

The study of conditions that lead to the temporary reemergence of problem behavior is of great importance. Although the temporary reemergence of more innocuous forms of problem behavior (e.g., tantrums) may not be of great concern, even one instance of more dangerous forms of problem behavior (e.g., eye gouging) can have serious and harmful consequences. Because of this, much work that has been conducted in the last decade to examine conditions that lead to the temporary reemergence of problem behavior, like resurgence and renewal. As a result of this work, the term “relapse” in behavior analysis has almost unanimously become associated with the study of *transient* forms of relapse. These forms of relapse are trigged by some transitional phase (e.g., reinforcement schedule thinning, introduction of different environmental stimuli), are transient in nature, and can be remedied with continued implementation of the behavior intervention with high procedural integrity. However, there are other conditions in which problem behavior may reemerge following a successful intervention, an important area of research that has largely been overlooked. The term “relapse” has also been used to refer to cases with *severe and prolonged* patterns of problem behavior that reemerge outside clinical settings, not under the direct observation of a clinician, and that are not remedied with continued implementation of an efficacious behavioral intervention.

Researchers have suggested or examined several conditions that may lead to more severe and *prolonged* patterns of problem behavior reemerging following successful intervention. For example, problem behavior may reemerge for prolonged periods of time if stakeholder adherence, or consistency, implementing the efficacious intervention is poor (i.e., rapid reacquisition). Likewise, prolonged patterns of problem behavior may emerge if there is a change in behavioral function after treatment (Brown et al., 2024; Lerman et al., 1994; Neely et al., 2024). For example, Lerman et al. (1994) used the term “relapse” to refer to four individuals whose caregivers reported the prolonged reemergence of problem behavior that necessitated readmission into a day treatment program designed to assess and treat severe problem behavior. In both admissions, researchers conducted a functional analysis to identify the environmental variables maintaining problem behavior for each client. For one client, the outcomes of the functional analysis were consistent across admissions. As such, the clinicians implemented the same treatment in the second admission as was implemented in the client’s first admission and observed reductions in problem behavior. It must be presumed that, for this client, caregiver nonadherence led to the reemergence of problem behavior between admissions. For the remaining three individuals, the FA conducted during the second admission was undifferentiated and researchers hypothesized that a change in behavioral function between admissions may have contributed to the prolonged reemergence of problem behavior.

Prolonged patterns of problem behavior may also emerge if treatment degradation occurs, not due to the implementer (e.g., habituation to a punishing stimulus; Ricketts et al., 1993). For example, Ricketts et al. (1993) conducted a long-term follow-up study for one individual who engaged in severe self-injury to monitor the ongoing effectiveness of treatment following successful intervention. From baseline to the end of the client’s intensive treatment, there was a 99.9% reduction in self-injury. Following this treatment period, clinicians conducted follow-up services with the client and monitored treatment effectiveness for 44 months. After 32 months of the treatment effectively maintaining very low rates of self-injury, there was an abrupt increase in self-injury that persisted over the next 12 months, despite consistent continued implementation of the treatment. As such, clinicians concluded that the once proven effective treatment, was in fact no longer effective. In this example, the reemergence of problem behavior was profound and prolonged across time (more than 12 months). As these results highlight, unlike more transient forms of the reemergence of problem behavior, these more prolonged forms are unlikely to resolve without additional intervention. However, few studies have empirically examined the conditions that lead to the prolonged reemergence of problem behavior or identified appropriate mitigation strategies. These forms of “prolonged relapse” appeared to be trigged by some change in the contingency maintaining problem behavior (e.g., caregiver nonadherence, change in behavioral function, habituation to a stimulus), resulting in the prolonged reemergence of problem behavior that required additional intervention and supports.

It is important to note that the reemergence of problem behavior following successful intervention (Kimball et al., 2023), applies to both transient *and* prolonged patterns of problem behavior reemerging. However, evolution of the conceptualization of “relapse” and nomenclature is needed to capture the differences between more *transient* and *prolonged* forms of problem behavior reemerging, particularly given these forms of “relapse” require different functional responses. Similar to how Jack Michael (1982, 1993) originally defined “establishing operation” and Laraway et al. (2013) later refined this framework by suggesting use of the term “motivating operations” as an umbrella term and “abolishing operation” and “establishing operation” as distinguished types of motivating operations. Likewise, this article introduces “lapse” and “relapse” as more nuanced terms to distinguish conditions under which problem behavior reemerges following successful intervention.

## Distinguishing “Lapse” and “Relapse”

With widespread acknowledgement of the reemergence of problem behavior following successful reductions (Kimball et al., 2023; Shahan, 2020), a more nuanced understanding is needed to distinguish between *transient* and *prolonged* reemergence of problem behavior. It is fortunate that the substance abuse literature provides some guidance on how we may reconceptualize our understanding of these two phenomena in the treatment of severe problem behavior. At first glance, the treatment of severe problem behavior and substance abuse may appear distinctly different. Although there are certainly some differences across these two disciplines, their similarities may be leveraged to better understand how to promote long-term maintenance of treatment outcomes. For example, both disciplines use operant contingency management interventions that are often highly efficacious at reducing the referral concern (Greer et al., 2016; Lussier et al., 2006; Madden, 2008; Rooker et al., 2013). The underpinnings of treatments for substance abuse and severe problem behavior are both firmly grounded in nonhuman and laboratory models of relapse (e.g., Carroll & Comer; 1996; Bigelow & Silverman, 1999; Fisher et al., 2022; Fisher et al., 2018; Greer & Shahan, 2019). Finally, “relapse” is common in both the treatment of substance abuse and severe problem behavior (Greer & Shahan, 2019; Kimball et al., 2023; Madden, 2008; Silverman et al., 2008; Silverman et al., 2023).

The substance abuse research distinguishes between temporary and prolonged patterns of relapse using the terms “lapse” and “relapse.” Lapses are temporally restricted or temporary patterns of previous behavior, whereas relapses are a more severe and prolonged pattern of previous behavior (Kaviyani et al., 2023; Mohseni et al., 2022). For example, Greenwald (2008) conducted a study examining the effectiveness of an abstinence reinforcement intervention to delay lapses in opioid use. In their study, a lapse was defined as return to “first use” whereas relapse was a return to “regular opioid use” (i.e., a prolonged and more severe pattern of unwanted behavior; p. 604). Likewise, other health disciplines have adopted the terminology “lapse” and “relapse” to refer to either a temporary or complete return to previous unhealthy behaviors, such as cardiovascular risk behaviors (e.g., diet, exercise, smoking; Bouton, 2000; Roordink et al., 2023). For example, if an individual who recently started to exercise two times a week for recommended weight loss returns to inactivity for 1 week, this may be considered a “lapse,” whereas if the individual experiences a series of lapses, such that there is a complete return to inactivity, this would be considered a “relapse" (Roordink et al., 2023).

Unlike substance abuse and health disciplines, terminology in the severe problem behavior research has yet to distinguish between conditions that result in a transient increase in problem behavior, relative to conditions that result in a sustained or prolonged increase in problem behavior. Although the term “relapse” has appeared in behavior analytic text to describe both phenomena, it has been conflated and there remains a lack of distinction between studies in which researchers examined conditions that resulted in a transient increase in problem behavior versus conditions that produced a prolonged increase in problem behavior. For example, many published studies on resurgence and renewal use the term “relapse” to refer to the transient increase in problem behavior that subsequently decreases with continued implementation of the efficacious intervention (e.g., Greer et al., 2023; Falligant et al., 2021; Falligant et al., 2024; Fuhrman et al, 2016; Saini et al., 2018; Saini et al. 2017; Mitteer et al., 2022; Weinsztok & DeLeon, 2022). To my knowledge, only one behavior-analytic study has used the terms “lapse” and “relapse” when referring to the reemergence of a behavior. Bouton (2000) used the terms “lapse” and “relapse” to describe the impact context has on learning new behavior, primarily via Pavlovian learning. Bouton describes some of the interdependency between “lapses” and “relapses” and the need to better understand mechanisms of learning behind both concepts; however, the article does not provide a definition of lapse or relapse. Rather, the reader must infer these definitions based on context. Nevertheless, across health and psychology disciplines there is general consensus that lapses are *temporally restricted and transient* whereas relapses are more pronounced and involve a *complete return of undesirable behaviors over an extended period*. Based on these definitions, the defining feature between “lapse’ and “relapse” is temporal (i.e., transient, temporary vs. prolonged, extended). The field of behavior analysis has much to contribute to create greater technological precision for these two concepts, given the field’s focus on identifying the influence of environmental events on behavior. In the treatment of severe problem behavior, a close analysis of cases in which problem behavior reemerges reveals different environmental events that led to either a transient or prolonged reemergence of problem behavior. “Lapses” in problem behavior occur following some change in the environment, such as a worsening reinforcement schedule or change in context. In terms of functioning, “lapses” can be mitigated by continued implementation of the efficacious treatment. In contrast, “relapses” in problem behavior occur due to some change in the contingencies governing problem behavior. Because of this, continued implementation of a previous treatment may not be effective.

Adopting the terminology “lapse” and “relapse” in the treatment of severe problem behavior to distinguish between the different conditions that led to the reemergence of problem behavior has many benefits. Doing so can create greater clarity on the distinct features of these two concepts, allow for a closer examination of the environmental conditions that may lead to “lapses” versus “relapses,” distinguish the clinical responses needed when “lapses” occur versus “relapses,” and provide an opportunity to examine how these two concepts may relate with one another. As such, the term “lapse” may be used to refer to temporary or transient increases in problem behavior that subsequently decrease with continued implementation of an efficacious intervention and “relapse” to refer to more severe and prolonged increases in problem behavior due to some change in the contingency maintaining problem behavior. These terms will be used accordingly for the remainder of this article.

## Independence and Interrelatedness of Lapse and Relapse

Although lapse and relapse are sometimes distinct concepts, they can and often are *interrelated.* Because caregivers are often responsible for implementing treatments for severe problem behavior, lapses may be particularly prone to become relapses if there is poor caregiver integrity or adherence. In particular, if lapses in problem behavior contact reinforcement, this can lead to relapse (i.e., rapid reacquisition). For example, the lapse of problem behavior from a context change (i.e., renewal) may turn into relapse if a stakeholder begins to consistently reinforce the problem behavior instead of continuing to implement the treatment. Stakeholder behavior is often pivotal to mitigate a lapse from becoming relapse. In fact, for socially maintained problem behavior lapses cannot evolve into relapse *independent of implementer errors.* In other words, regardless of the condition that led to the reemergence of problem behavior (e.g., a worsening of reinforcement conditions, a change in context), this will be a transient phenomenon *as long as the intervention continues to be implemented consistently as designed.* However, caregivers and other implementers often do not implement interventions consistently or as designed in the face of these challenges (Mitteer et al., 2018; Williams et al., 2023), increasing the likelihood of a lapse becoming a complete relapse in problem behavior.

Perhaps because of the importance of implementer behavior in preventing lapses in problem behavior from becoming relapses, the two phenomena have largely become recognized as interdependent or the same phenomena in which (1) lapses must precede a relapse and (2) implementer behavior mediates whether a lapse becomes a relapse. Although this may be true for some lapse conditions, as outlined above, lapse and relapse can be distinct phenomena*.* In other words, lapses may not always precede a relapse, and relapses can occur independent of implementer error.

Consider, for example, how relapse may occur without a prior lapse in problem behavior. If problem behavior reemerges from treatment degradation not due to implementer integrity or adherence, such as the case in Ricketts et al. (1993), this relapse of problem behavior was not preceded by a lapse in which there is a temporary increase in problem behavior due to a transitional condition (e.g., worsening of reinforcement conditions, change in context). In addition, the reemergence of problem behavior occurred independent of the implementer’s integrity and/or adherence to the treatment procedure. Thus, in this example, relapse was not proceeded by a lapse and is not mitigated by an implementer’s continued implementation of an efficacious intervention, demonstrating the independence of lapse and relapse phenomena.

Automatically maintained behavior is another example of how a lapse in problem behavior can sometimes evolve into relapse independent of implementer behavior. Bouton (2000) describes a hypothetical smoker whose initial lapse is the result of a change in context such as entering a bar (renewal). This context change introduces additional contextual cues (e.g., drinking alcohol) that further act on the smoker influencing their behavior to smoke again. Bouton describes this as, “. . . an expanding renewal effect that is created by an ever-expanding set of contextual cues. In this way, a slip or lapse may spiral into relapse” (p. 60). This lapse into relapse phenomena, independent of implementer behavior, can only occur if the behavior in and of itself produces reinforcement, as is the case of smoking. Thus, in the treatment of severe problem behavior, we would not expect a lapse of socially mediated problem behavior, despite a large set of contextual cues, to spiral into relapse independent of implementer behavior. However, this lapse into relapse phenomena that is independent of implementer behavior may occur in cases in which a client’s problem behavior is automatically maintained. For example, Falligant et al. (2024) conducted a retrospective CCCS to examine renewal of automatically maintained problem behavior based on a series of context changes (e.g., person, location). They found renewal of automatically maintained behavior occurred for approximately two thirds of participants, which is consistent with previous findings (cf. Muething et al., 2022). Of relevance to this lapse into relapse independent of implementer behavior phenomena, studies that have examined renewal of automatically maintained problem behavior have found that in some cases the initial increase in problem behavior (lapse) did not always decline in an orderly fashion as is always seen with renewal of socially maintained problem behavior (Falligant et al., 2021; Muething et al., 2020). Thus, if automatically maintained problem behavior remerges due to a change in condition (i.e., lapses), problem behavior may spiral into a relapse independent of the implementer’s behavior due to the reemerged problem behavior introducing additional contextual cues that further influence the occurrence of problem behavior. Future research could continue to explore the extent to which lapses in automatically maintained behaviors may be more prone to spiral into relapses, independent of implementer behavior, due to an expanding renewal effect.

## “Lapse” and “Relapse” in Research and Clinical Practice

### Research Implications

In delineating the concepts of lapse and relapse within the assessment and treatment of severe problem behavior, we see there is a large and growing body of research related to conditions that lead to lapses in problem behavior, but little research on the conditions that lead to relapse in problem behavior. Several conditions have been identified that lead to lapses in problem behavior including resurgence, renewal, reinstatement, and spontaneous recovery (Wathen & Podlesnik, 2018). We also know that some conditions that lead to lapses in problem behavior are more common in clinical settings than others, such as resurgence and renewal (Kimball et al., 2023). The explosive growth in our identification and understanding of the conditions that lead to transient increases in problem behavior has generated clinical guidelines for how to mitigate these lapses, which will be discussed below. Finally, several studies have demonstrated the importance of caregiver behavior in mitigating lapses from becoming relapses (e.g., Brown et al., 2020; Mitteer et al., 2018; Mitteer et al., 2021; Williams et al., 2023). Despite this, few studies have examined how to increase caregiver persistence of appropriate behavior under these treatment challenges. As such, this will be an important area of future research to mitigate caregiver-mediated lapse-to-relapse phenomena (i.e., rapid reacquisition).

Relative to the large and growing body of research examining conditions that lead to lapses in problem behavior, the study of conditions that lead to relapse of problem behavior is in its infancy. Known relapse conditions include caregiver nonadherence, a change in behavioral function, and treatment degradation not due to implementer. Of the three different conditions that have been identified to lead to relapse of problem behavior, empirical studies examining these conditions remain scarce. Much of the published text on relapse is from several decades ago and limitations of these texts prohibit much generalizable knowledge for us today. For example, many of these texts only contain anecdotal case reports of individuals who engaged in severe forms of self-injury and whose treatments predated functional analysis methodology and relied on punishers in the form of contingent shocks (e.g., Bruhl et al., 1982; Schroeder et al., 1982). Given these limitations, the findings of these studies are not highly generalizable to other forms of severe problem behavior (e.g., aggression) or function-based interventions that largely rely on reinforcement to promote behavior change. Given this, the overall prevalence or likelihood of any of the conditions leading to relapse remains largely unexplored.

One recent exception is Brown et al. (2024), who examined the prevalence of change in behavioral function contributing to relapse. Although researchers have called attention for decades to the possibility of change in behavioral function contributing to relapse (e.g., Carr & McDowell, 1980; Lerman et al., 1994; Wunderlich et al., 2020), Brown et al. is the first study to empirically examine the prevalence of this phenomenon. In their retrospective CCCS, they examined FA outcomes to identify if a change in behavioral function occurred in cases that were readmitted for intensive treatment services due to reported relapse of problem behavior. Out of the 22 cases in their study, they found a novel behavioral function emerged between admissions for approximately 20% of cases, providing the first empirical evidence that a change in behavioral function can be a contributing factor to relapse of problem behavior outside clinical settings. However, these findings need to be replicated and extended prior to any generalizable conclusions about change of behavioral function as a contributing factor to relapse. In addition, research should explore the mechanisms behind the emergence of a novel behavioral function. For example, a novel behavioral function could emerge from exposure to a novel establishing operation (e.g., client gets a new highly preferred tangible) and/or a communication deficit (e.g., client enters school and does not have a communication response to request a break). As an alternative, a behavioral function could emerge through a process of conditioned reinforcement if the client’s problem behavior was at least in part maintained by automatic reinforcement (cf. Rooker et al., 2011, as an example). Finally, it is possible that stakeholders could contribute to the emergence of a novel behavioral function. It is worth noting that in these situations caregiver behavior may not always represent a reduction in treatment integrity if they have not received prior training on that skill. For example, imagine a situation in which a caregiver was trained to implement a treatment to decrease problem behavior maintained by access to attention by withholding attention following problem behavior and providing attention contingent on appropriate requests for attention. Although the caregiver continues to implement the attention treatment with high procedural integrity, the child starts school resulting in an increase in the establishing operation for the child to escape demands. The child begins to engage in problem behavior in this novel context and the caregiver provides escape following the problem behavior. Thus, despite continued high procedural integrity on the recommended treatment for attention-maintained problem behavior, a novel behavioral function may emerge.

There may be several reasons for the disparity in the amount of research examining conditions that lead to lapses versus relapses in problem behavior. First, a lack of distinction may have obscured the study of these two distinct phenomena. In providing this distinction, future research can examine conditions that lead to lapses and relapses, as well as explore the relation between these phenomena. Another potential reason for the disparity between the amount of lapse and relapse research is that studying the conditions that lead to lapses versus relapses may require different methodologies. Models designed to examine lapses in problem behavior (e.g., resurgence, reinstatement) may not be as suitable to examine relapse phenomena. Rather, lapse models have been designed to simulate specific environmental events that lead to the reemergence of behavior. Conditions that promote lapses in problem behavior can often be examined with relative ease within clinical settings given many of these conditions, although not all, occur during or shortly after treatment. As a result, studies that examine lapses in problem behavior are well-suited to be conducted in highly controlled clinical settings using standard single-subject designs and continuous direct observation measures. In contrast, it is often challenging to examine conditions that lead to relapse of problem behavior in highly controlled clinical settings. Many of the conditions that lead to relapse (e.g., transfer of behavioral function, habituation to a stimulus) are likely to occur several months to years after severe behavior treatment services are completed, making it more challenging to use single-case designs and continuous direct observation measures. In addition to single-case designs, researchers should consider the use of longitudinal or CCCS methodologies (Hagopian, 2020) to examine conditions of relapse. Finally, relapse research may be more likely than lapse research to rely on the use of nondirect measures that can easily be administered outside clinical settings (e.g., permanent products, caregiver reports). Acknowledging the different forms of measurement and study design that will likely be needed to examine lapses and relapses in problem behavior, as well as identifying reliable and accurate forms of nondirect measures, will be important to further the study of relapse.

### Clinical Implications

In experimental preparations, researchers control the environmental events and contingencies surrounding problem behavior allowing them to draw causal conclusions about lapse and relapse phenomena. However, when problem behavior reemerges outside of experimental settings the result is that clinicians are unaware of the environmental events influencing the reemergence of problem behavior. As such, a foremost step to identify if a lapse verses relapse has occurred is to conduct an assessment to identify the environmental conditions surrounding problem behavior and generate hypotheses on potential controlling variables. To accomplish this, clinicians could conduct multiple observations of the stakeholder implementing the treatment with the client during which the clinician collects procedural integrity and treatment efficacy data (i.e., data on how well a treatment works in real-world settings when implemented by natural incumbents). If integrity and efficacy data are high across multiple data points, this could suggest a lapse in problem behavior, particularly if there is some evidence of a recent environmental change (e.g., time away from treatment, implementation in a new setting). If stakeholder procedural integrity data are low, clinicians will want to intervene to improve stakeholder integrity (i.e., the accuracy with which the stakeholder is implementing the treatment) to observe if this once again produces decreases in the reemerged problem behavior. If improving integrity results in a decrease in problem behavior, clinicians can conclude that a lapse in problem behavior occurred (i.e., reinstatement). If procedural integrity data are high, but efficacy data are low (i.e., the client is engaging in elevated rates of problem behavior), this may be indicative that a relapse in problem behavior has occurred. In these situations, clinicians should work to identify if a change in the contingency governing problem behavior has occurred.

Lapses and relapses in problem behavior often necessitate different responses from clinicians in clinical practice. Table 1 outlines lapse and relapse conditions as well as mitigation responses for each. We know from the growing body of research examining lapses in problem behavior that clinicians should anticipate for lapses at key points in time (e.g., schedule thinning), preemptively use lapse-mitigation strategies to inoculate the amount of problem behavior that reemerges, and educate caregivers and other stakeholders that lapses are going to occur and provide support during these times (e.g., generalizing to a novel setting). Because lapses are likely to occur, clinicians should proactively work with patients and stakeholders to prepare for and mitigate the amount of problem behavior that reemerges (Kimball et al., 2023). Lapses in problem behavior, although they can be alarming for stakeholders who are not anticipating temporary increases in problem behavior, are common and to be expected during and shortly after treatment of severe problem behavior. When lapses occur, the general mitigation response is to continue implementing the treatment with high procedural integrity (Neely et al., 2024). If stakeholder procedural integrity is not optimal, clinicians may also need to conduct additional caregiver training. However, in some cases, such as for individuals with severe forms of problem behavior in which even one instance of problem behavior can produce substantial harm, clinicians will want to consider addition lapse mitigation responses. Fortunately, there are several evidence-based strategies to mitigate lapses in problem behavior. For example, using discriminative stimuli to signal periods of extinction and reinforcement (e.g., Fuhrman et al., 2016; Fisher et al., 2020) and using preferred communication modalities to strengthen persistence of the communication response in the face of treatment challenges (Ringdahl et al., 2018). In addition, clinicians may consider regressing to denser schedules of reinforcement, reducing response effort of an alternative response (e.g., a functional communication response), or increasing the density of the prompt schedule for alternative behavior. We encourage readers to see Neely et al. (2024) for a comprehensive support guide for clinicians on how to respond to lapses in problem behavior.

**Table 1:** Types of Lapses, Relapse, and Mitigation Responses

Lapse	Definition	General Mitigation Response
Resurgence	The temporary reemergence of problem behavior due to a worsening in reinforcement conditions ^a^	Continue implementing treatment as prescribed
Renewal	The temporary reemergence of problem behavior due to change in environmental context ^b^	Continue implementing treatment as prescribed
Reinstatement	The temporary reemergence of problem behavior due to the independent or dependent delivery of a reinforcer that previously maintained problem behavior ^b^	Continue implementing treatment as prescribed (independent) or conduct additional training with caregivers to increase procedural integrity (dependent)
Spontaneous Recovery	The temporary reemergence of problem behavior due to “time off” from treatment following extinction of the problem behavior ^b^	Continue implementing treatment as prescribed
Relapse	Definition	General Mitigation Response
Change in Behavior Function	The prolonged reemergence of problem behavior due to a change in the maintaining variable(s) ^c^	Initiate treatment for newly emerged function of problem behavior
Treatment Degradation	The prolonged reemergence of problem behavior from treatment degradation not due to implementer ^d^	Examine other potential efficacious treatments
Poor Adherence	The prolonged reemergence of problem behavior due to implementers not consistently and/or accurately implementing the recommendation treatment ^e^	Conduct additional training (procedural integrity) and identify barriers affecting ongoing implementation in the home and community (adherence)

*Note.* ^a^ Greer & Shahan, 2019; ^b^ Wathen & Podlesnik, 2018; ^c^ Brown et al., 2024; ^d^ Ricketts et al., 1993; ^e^ St. Peter, 2023. The mitigation responses listed are general responses and not comprehensive (cf. Neely et al., 2024, and Kimball et al., 2023, for a more comprehensive guide on mitigation strategies for lapses in problem behavior)

In contrast, relapse of problem behavior is not anticipated and should serve as a signal to stakeholders and clinicians that additional or more intensive supports are once again needed. In cases of relapse, clinicians will likely need to conduct additional analyses to determine the cause of relapse. This may include assessing if there are barriers affecting stakeholder adherence (i.e., their ability to consistently implement the treatment with accuracy; cf. Moore & Amado, 2021), conducting a functional analysis to determine if a change in behavioral function has occurred (e.g., Brown et al., 2024), or if a new treatment is needed to decrease problem behavior.

### On Terms

Other terms have been suggested to describe the reemergence of problem behavior such as “reoccurrence” (e.g., Lattal & Wacker, 2015), “recidivism”, and “treatment relapse." The terms “recidivism” and “treatment relapse” continue to be used, particularly in vernacular, when describing cases in which problem behavior reemerges. Given this, this article will briefly address why these terms should not be adopted in lieu of “relapse” or “lapse” to describe cases in which problem behavior has reemerged (cf. Shahan, 2020, for a discussion on the term reoccurrence).

The term “treatment relapse” has been used as an umbrella term to refer to the reemergence of problem behavior or other clinically concerning behaviors that appeared to have been previously treated (e.g., Kimball et al., 2023; Lerman et al., 1994; Mace & Nevin, 2017; Pritchard et al., 2014; Wathen & Podlesnik, 2018). When we use the terms “relapse” or “lapse,” we are referring to an individual’s return to some previous problematic behavior (e.g., drug use, problem behavior, physical inactivity). Thus, using the term “treatment relapse,” which suggests one is relapsing to the treatment not to the previously problematic behavior, lacks technological precision. As such, it is more technologically precise for the field moving forward to refrain from including the term “treatment” when discussing cases of “relapse” and “lapse.”

The term “recidivism” is also sometimes used when describing relapse of problem behavior that necessitates additional intensive treatment services. The term “recidivism” is used in broader psychology to refer to the reoccurrence of delinquent behavior exhibited by juvenile or criminal offenders (American Psychological Association, n.d.). This definition of “recidivism” is not consistent with what behavior analysts are describing when they use the term to refer to individuals with intellectual and/or developmental disabilities whose problem behavior reemerges following successful intervention. As such, limiting our use of the term “recidivism” to the reoccurrence of illegal behavior will prevent someone from misconstruing that behavior analysts liken individuals with intellectual and/or developmental disabilities who reengage in severe forms of problem behavior to be the same as neurotypical repeat criminal offenders. For these reasons, the term “relapse” is a more appropriate term to describe relapse of problem behavior that necessitates additional intensive treatment services.

## Summary and Future Directions

Continued research in lapse and relapse is paramount to optimize the long-term health outcomes for individuals with intellectual and developmental disabilities who engage in severe forms of problem behavior. This article outlines the need for greater technological distinction between “lapse” and “relapse.” As our science and clinical practices evolve, so too should our use of terms (Carr & Briggs, 2011). With greater distinction between lapse and relapse phenomena, there are clear pathways for future research. First, although there is a growing body of research on lapses in problem behavior, some conditions have remained largely unexamined in clinical populations (e.g., spontaneous recovery, Kimball et al., 2023; mitigation of reinstatement, Kranak et al., 2022) and many mitigation strategies have mixed evidence that precludes clear best-practice guidelines (Kimball et al., 2023). Second, the near complete absence of research on conditions that lead to relapse in problem behavior makes this a dire area for future research. Finally, it is important that future research identifies ways stakeholders (e.g., clinicians, researchers, caregivers) can readily ascertain if a lapse has occurred versus relapse. At present, if problem behavior reemerges following successful intervention clinicians are likely using informal interviews with stakeholders to try and identify if any lapse conditions are present (e.g., worsening of reinforcement condition, change in context, errors of commission or omission), as well as highly suspected relapse conditions (e.g., adherence). Then, only after prolonged periods of time with continued problem behavior are clinicians considering that relapse may have occurred. Although this approach may be sufficient in some circumstances, it may be problematic for several reasons. For example, although caregivers can be accurate reporters when they observe large changes in the frequency of their child’s problem behavior (Becraft et al., 2023), it is unclear the extent to which they would be able to accurately report their own adherence to interventions or identify conditions that may promote relapse of problem behavior (e.g., identifying a change in behavioral function). Likewise, if relapse has occurred, quick identification and intervention can help mitigate additional injury and harm to the client and others. With a more nuanced understanding of how the concepts of lapse and relapse are often related, yet are distinct, behaviors analysts will be uniquely positioned to advance our understanding and science of designing durable treatments the withstand the test of time and environmental disruptors.

## Data Availability

Data sharing is not applicable to this article as no datasets were generated or analyzed.
